# Splicing defect in *FKBP10* gene causes autosomal recessive osteogenesis imperfecta disease: a case report

**DOI:** 10.1186/s12881-018-0579-8

**Published:** 2018-05-25

**Authors:** Fatemeh Maghami, Seyed Mohammad Bagher Tabei, Hossein Moravej, Hassan Dastsooz, Farzaneh Modarresi, Mohammad Silawi, Mohammad Ali Faghihi

**Affiliations:** 1 Department of Medical Genetics, Medical School, Shiraz University of Medical Sciences, Shiraz, Iran; 2 Pediatric Department, School of Medicine, Shiraz University of Medical Sciences, Shiraz, Iran; 3 Persian BayanGene Research and Training Center, Dr. Faghihi’s Medical Genetics Center, Shiraz, Iran; 4 Center for Therapeutic Innovation, Department of Psychiatry and Behavioral Sciences, University of Miami Miller School of Medicine, 1501 NW 10th Ave, BRB 508, Miami, FL 33136 USA

**Keywords:** Osteogenesis imperfect (OI), *FKBP10*, Novel splice mutation, Case report

## Abstract

**Background:**

Osteogenesis imperfecta (OI) is a group of connective tissue disorder caused by mutations of genes involved in the production of collagen and its supporting proteins. Although the majority of reported OI variants are in *COL1A1* and *COL1A2* genes, recent reports have shown problems in other non-collagenous genes involved in the post translational modifications, folding and transport, transcription and proliferation of osteoblasts, bone mineralization, and cell signaling. Up to now, 17 types of OI have been reported in which types I to IV are the most frequent cases with autosomal dominant pattern of inheritance.

**Case Presentation:**

Here we report an 8- year- old boy with OI who has had multiple fractures since birth and now he is wheelchair-dependent. To identify genetic cause of OI in our patient, whole exome sequencing (WES) was carried out and it revealed a novel deleterious homozygote splice acceptor site mutation (c.1257-2A > G, IVS7-2A > G) in *FKBP10* gene in the patient. Then, the identified mutation was confirmed using Sanger sequencing in the proband as homozygous and in his parents as heterozygous, indicating its autosomal recessive pattern of inheritance. In addition, we performed RT-PCR on RNA transcripts originated from skin fibroblast of the proband to analyze the functional effect of the mutation on splicing pattern of *FKBP10* gene and it showed skipping of the exon 8 of this gene. Moreover, Real-Time PCR was carried out to quantify the expression level of *FKBP10* in the proband and his family members in which it revealed nearly the full decrease in the level of *FKBP10* expression in the proband and around 75% decrease in its level in the carriers of the mutation, strongly suggesting the pathogenicity of the mutation.

**Conclusions:**

Our study identified, for the first time, a private pathogenic splice site mutation in *FKBP10* gene and further prove the involvement of this gene in the rare cases of autosomal recessive OI type XI with distinguished clinical manifestations.

**Electronic supplementary material:**

The online version of this article (10.1186/s12881-018-0579-8) contains supplementary material, which is available to authorized users.

## Background

Osteogenesis imperfecta (OI) (also known as brittle bone disease) is a heterogeneous group of connective tissue disorders characterized by easy and frequent fractures in long bones leading to malformation, disability and wheelchair dependence [1]. Other less frequent symptoms are short stature, bluish sclera, dentinogenesis imperfecta and hearing impairment [2]. Clinical presentations of the disease range from mild asymptomatic forms with an increased tendency to fracture to more severe neonatal forms with early death due to multiple bone and spine fractures [3].

The David Sillence’s classification of OI included types I to IV [4]; but, now this classification has been extended to XVII groups. The inheritance pattern of types I to V is autosomal dominant but types VI to XVII which are the rare forms of the disease are presented with autosomal recessive inheritance [5–7].

The mutations responsible for OI types I, II, III, and IV which are the most common form of OI (85–90%) occur in either the *COL1A1* or *COL1A2* gene, encoding type I collagen [3, 8–10]. But, mutations in other genes are accounted for other types of the disease, presenting 10–15% of all cases with autosomal recessive inheritance [11] (Additional file 1: Table S1). OI incidence has been reported as high as 1/15,000 to 1/20,000 live births [12] but in Iranian population the frequency has not been estimated yet.

The aim of current study was to identify disease-causing mutation in an affected boy with OI without prior history of OI in his parents and extended family members.

## Case Presentation

The proband is an 8–year-old Iranian boy who was born to consanguineous marriage partners (first-degree cousins) by full-term normal vaginal delivery (NVD). He had birth weight of 3400 g, birth length of 50 cm and head circumference of 35 cm. It is worth noting that the first fracture was observed in elbow during delivery and other fractures were seen at the age of 2 and 5 months in other long bones, followed by several fractures in all bones. After receiving intravenous Pamidronate, every 3 months, number of fractures reduced from 7 to 8 per year to only 2 per year. The intellectual capacity was intact and cognitive function was in normal range. Clinical findings were also observed as blue sclera, which was diminished with age. Hearing loss, dental eruption, and symptoms of contracture deformity (reduced ability to move an area of his body) have not been observed in the patient. Echocardiography was performed which showed normal heart function. Also, abdominal ultrasonography revealed normal size of liver and spleen. He is currently wheelchair dependent and able to take few steps using walker stand. Now, he has height of 102 cm (− 4.3 SD), head circumference of 47.5 (10th centile) and weight of 14,600 g (25th centile). Repeated radiologic reports were in favor of OI with reduced bone density and multiple fractures in various stages of healing. Laboratory tests were also performed for the proband which revealed following results: increased levels of calcium (10.7 mg/dl, normal range: 8.8–10.2 mg/dl) and sodium (139 mEq/L, normal range: 6–20 mEq/L), but normal levels of phosphorus (5.8 mg/dl, normal range: 4–6.5 mg/dl), potassium (4.1 mEq/L, normal range: 3.5–5 mEq/L), and alkaline phosphatase (650 unit/liter, normal range: 180–1200 U/L). Also, thyroid and kidney function tests were reported in normal range.

The mother of proband was also pregnant and prenatal diagnosis test on chorionic villus sample (CVS) showed that fetus had the same pathogenic mutation like the proband. Therefore, the pregnancy was terminated therapeutically at the 18 weeks of gestation.

### Whole Exome Sequencing (WES)

To investigate disease causing mutation in our patient, WES was performed to amplify and sequence all exons of protein-coding genes as well as some other important genomic regions. In our study, next generation sequencing was carried out to sequence close to 42 million reads on Ion Proton Sequencer. In general, test platform examined > 95% of the targeted regions with sensitivity of above 99%. In this test, point mutations and micro-insertion/deletions and duplication (< 20 bp) can be simultaneously identified. Bioinformatics analysis of the sequencing results was performed using BWA aligner [13], GATK [14] and Annovar open access software [15] as well as public databases and standard bioinformatics software. Sequences text files were aligned using BWA aligner tool, variants were identified using GATK and then annotated utilizing annovar software.

### Sanger Sequencing

To confirm the presence and the pattern of inheritance of a novel identified mutation, peripheral blood samples were obtained from proband, his parents, grandparents from both sides, and other extended family members. All samples were processed for DNA extraction using QIAamp DNA Blood Mini Kit (Cat No./ID: 51104, Germany) according to the manufacturer’s instructions. Moreover, DNA of CVS sample was extracted using QIAamp DNA Mini Kit (Cat No./ID: 51304, Germany). Following primers were used to amplify exon eight of *FKBP10* gene as well as its flanking intronic sequences to look for the mutation: FKBP10_E8_F: ccaagtcaccagtgggagta and FKBP10_E8_R: ttatggtctcagcccctcac. Amplified DNA was sequenced from both directions using Sanger Sequencing kit (ABI BigDye Terminator Cycle Sequencing Kit, Applied Biosystems®, USA) according to the company protocol. Then, Sanger sequencing data was analyzed using NCBI BLAST and 4Peaks free software.

### RT-PCR

In current study, commercially available cDNA from various human tissues were analyzed to identify the tissues with expression of *FKBP10* gene. Also, RNA was extracted from peripheral blood and skin biopsy samples (the skin fibroblast culture conditions is given in Additional file 1) of the proband and his parents using TriPure (Additional file 1) and RNeasy Mini Kit (QIAGEN, Germany), respectively. RNA concentration, purity, and integrity were assessed using NanoDrop ND1000 spectrophotometer (Thermo Fisher Scientific) and electrophoresis of the 2 μL extracted RNA on 2% agarose gel containing SYBR Safe™ DNA gel stain (Invitrogen) under the ultraviolet (UV) light. The isolated RNA was stored at − 20 °C until use. Then, they were subjected to cDNA synthesis using SuperScript First-Strand Synthesis System for RT-PCR (Invitrogen, USA) according to the standard company protocol. The resulted cDNA samples were used for performing RT-PCR using primers spanning exon 7, 8 and 9 of *FKBP10* transcript (Sense-Primer: 5’gaactggagacaagatccct 3′ and Antisence-Primer: 5′ cttgatgaaggtggagaactc 3′, Product: 526 bp) and then the results were compared to normal cDNA sequence for identification of any changes in the transcript splicing events.

### Quantitative Real Time PCR

Real-Time PCR using PowerUp SYBR Green Master Mix (ABI, USA, Cat number: A25918) on Applied Biosystems 7500 Real-time PCR System was performed in triplicate to investigate any reduction in *FKBP10* gene expression in the skin biopsies from the proband and his parents compared to normal controls (Additional file 1). In this study, human Glyceraldehyde 3-phosphate dehydrogenase (*GAPDH*) was used as reference gene. Following Exon-Exon Junction primer pairs were used in our Real-Time PCR for the interested gene (F-QPCR- FKBP10*:* gttcacctcgcatgactac and R-QPCR- FKBP10: cctctctcccacacacat) and GAPDH gene (F-QPCR-GAPDH: gctctctgctcctcctgttc and R-QPCR-GAPDH: cgaccaaatccgttgactcc).

## Results

NGS result revealed that the proband was homozygous for a splice-acceptor mutation in *FKBP10* gene (FKBP10–201: ENST00000321562.8, NM_021939.3: c.1257-2A > G, IVS7-2A > G). This novel homozygous variant is located in position 39,977,197 on chromosome 17q21.2 close to exon 8 in *FKBP10* gene. It is predicted that this splice site change can lead to a frameshift mutation in FKBP10 protein (FKBP10–201: ENST00000321562.8, NM_021939.3: p.H420PfsX12). All information about this mutation and all bioinformatics software which were used to predict the functional effects of this mutation are given in Additional file 1: Table S2. This mutation is predicted to be pathogenic using MutationTaster tool and highly conserved using Phastcons and GERP program (Additional file 1: Table S2). Allele frequency for this mutation is not available in ExAC (http://exac.broadinstitute.org/), GnomAD (http://gnomad.broadinstitute.org/), Kaviar (http://db.systemsbiology.net/kaviar/cgi-pub/Kaviar.pl), GME (http://igm.ucsd.edu/gme/), ESP (http://evs.gs.washington.edu/EVS/), 1000Genomes (http://www.internationalgenome.org/1000-genomes-browsers) and our database (1000 Iranian whole exome sequencing data as BayanGene). There is no report in literatures on pathologic manifestation of this mutation and its phenotype relation to the OI type XI or Bruck syndrome, so this mutation is considered as novel variant.

**Fig. 1:**
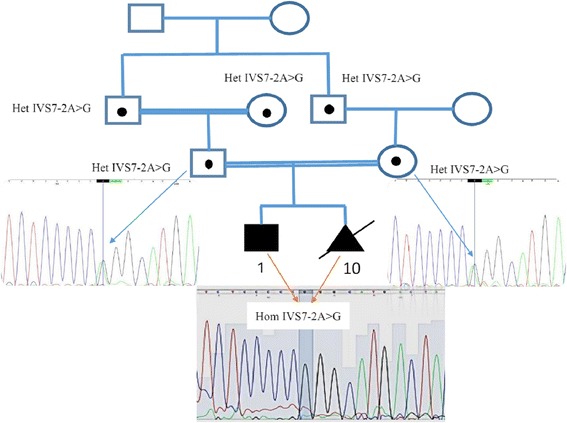
Pedigree and Sanger sequencing details. Using Sanger sequencing, the inheritance mode of autosomal recessive was confirmed in this family based on identified homozygote mutation in the proband and heterozygote mutation in his parents and three of his grandparents. Here only sequencing graph from the proband and his parents are given. Het: Heterozygous. Hom: Homozygous

Sanger sequencing results revealed that proband and fetus were homozygous for this new mutation but both his mother and father as well as three of his grandparents were heterozygous for the mutation (Fig. 1), indicating the autosomal recessive pattern of inheritance for this disease. Also, it confirmed that the observed genotype in the proband, his parents, and extended family members segregates with the disease phenotype.

**Fig. 2:**
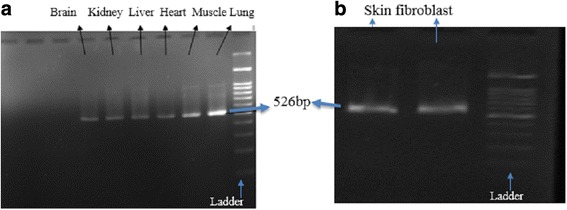
**a**). RT-PCR for identification of *FKBP10* gene expression in several commercially available cDNAs from various human tissues. It can be seen that the expression of this gene is observed in brain, liver, kidney, heart, muscle, and lung. **b**). RT-PCR shows the presence of FKBP10 transcript in skin biopsy samples in the normal indivitual

**Fig. 3:**
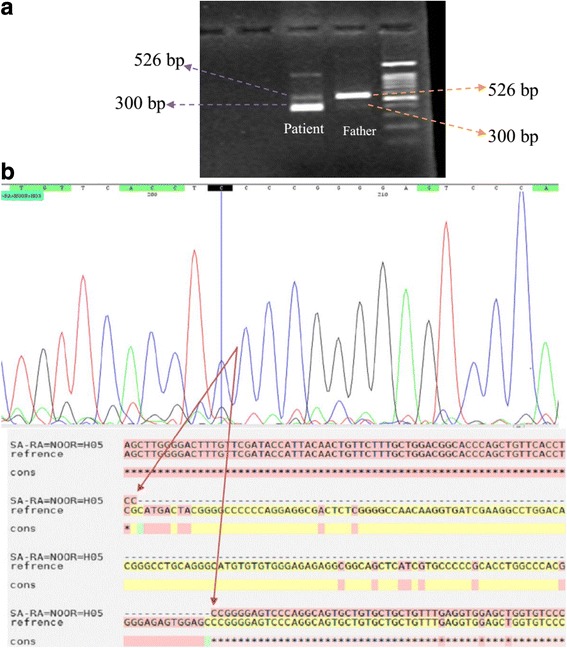
**a**). RT-PCR shows exon 8 skipping which produces a sharp skipped product of 300 bp and a week normal PCR band of 526 bp in the proband but in his father, a 526 bp with sharp PCR band and 300 bp with weak PCR band is detected. **b**). Sequencing data of skipped product (300 bp) from proband. Data was blasted to reference FKBP10 transcript and confirmed the exon 8 skipping. (*) symbol: Cons: conserved nucleotide in the blast. (−) symbol: deleted nucleotide

Moreover, RT-PCR analysis of commercially available cDNA from lung, brain, muscle, kidney, heart, and liver and also skin biopsy samples from patient showed the presence of FKBP10 transcript (Fig. 2a, b). As can be seen in Fig. 3a, RT-PCR showed exon 8 skipping since it produced a sharp PCR band of 300 bp (skipped product) and a week PCR band of 526 bp (normal product) in the proband compared to 526 bp with sharp PCR band and 300 bp with weak PCR band in a normal heterozygous sample. In addition, Sanger sequencing from the 300 bp product provided from the proband confirmed the exon 8 skipping (Fig. 3b).

**Fig. 4:**
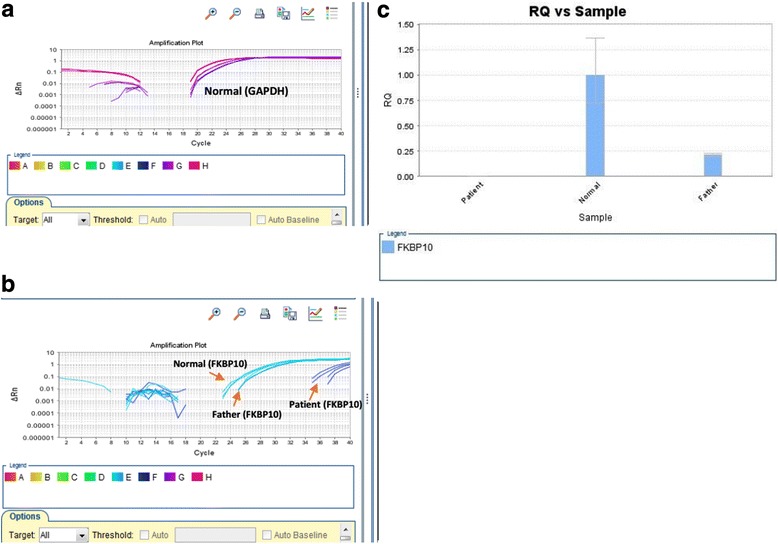
Real-Time analysis of *GAPDH* and *FKBP10* gene in the patient, his father and normal individual. **a**). As seen in this figure, *GAPDH* expression shows same levels for the patient, his father and normal individual **b**). Comparison between expression levels of *FKBP10* shows different levels in the proband, heterozygous individual and normal individual **c**). Relative quantification (RQ) result. It shows that normal individual (the calibrator) has a RQ value of 1 meaning no changes in its expression level but in the proband RQ is zero which indicates no expression of *FKBP10*. In his father, the gene is 75 times less expressed

Real-Time PCR on skin biopsies from the proband and his father clearly showed nearly the full depletion in the level of *FKBP10* expression in the proband and around 25% expression of *FKBP10* in his parents compared to normal controls, confirming exon 8 skipping and frameshift truncation of FKBP10 protein (Fig. 4 a-c). As can be observed in the Fig. 4b, a leaky expression of *FKBP10* is seen in the proband with both mutated copies of *FKBP10* gene. Therefore, segregation studies using Sanger sequencing, RT-PCR, and Real-Time PCR analysis confirmed the pathogenicity of this splice site mutation in *FKBP10* gene.

## Discussion and Conclusions

OI is a heterogeneous disease resulted from disease-causing mutations in different genes contributing to 17 different types of OI [2, 16]. Type XI OI is resulted from disease causing mutations in *FKBP10* gene. This gene is located on long arm of chromosome 17 (17q21.2) consisted of 10 exons which is encoded for a highly conserved family of intracellular proteins called immunophilins [17, 18]. This protein is essential for proper formation and balance between elastin and collagen molecules presented in the extracellular matrix [19]. Also, this protein acts as molecular chaperon and have important roles in collagen biosynthesis and folding [19, 20]. Cross linking between collagen molecules creates durable collagen molecules which resist fractures. It should be noted that an important role of FKBP10 protein can be considered in the hydroxylation of collagen prior to cross link formation [20]. Therefore, any impaired FKBP10 protein can be deleterious in the establishment of durable collagen molecules.

For the first time, Alanay et al. in 2010 reported that a mutation of *FKBP10* gene is involved in OI type XI [3]. They studied a cohort of five consanguineous Turkish families, and also a Mexican-American family with moderately severe recessively inherited OI. They identified a 4 Mb region, a locus for OI, on chromosome 17 spanning *FKBP10* gene and then it was reported as a candidate gene for OI type XI. They sequenced all ten exons of *FKBP10* gene using Sanger sequencing technique to detect the causative gene in their study.

Since the first report, several mutations have been identified in *FKBP10* gene. It is worth to note that homozygous mutations in *FKBP10* have been previously reported in OI type XI (OMIM number 610968), an autosomal recessive form of OI. In addition, mutations in *FKBP10* have been found in Bruck Syndrome 1 (OMIM number 259450), a musculoskeletal disorder with some overlapping phenotypes with OI. In our study we identified a private splicing mutation in this gene using next generation sequencing which caused its exon 8 skipping, resulting to a frameshift mutation and truncation of FKBP10 protein. As described in the result section, this mutation can cause leaky expression of *FKBP10* gene but the few produced transcripts are not sufficient to prevent the clinical abnormalities in the patient. In addition, in the heterozygous individual we expected to observe around 50% decrease in the expression levels of this gene but nearly 25% expression of this gene was detected without any clinical findings in the heterozygous case. Current study uncovered that this mutation is pathogenic in our patient affected by OI type XI. Therefore, our study is in accordance with study conducted by Alanay et al. in 2010 [3] and can be considered as OI type XI with moderate to severe and progressive nature of phenotype.

As mentioned above, the splicing mutation found in our study causes the production of mRNA molecule with lack of exon 8 producing truncated FKBP10 protein without the last PPIase and HEEL domains. In addition, defects in FKBP10 lead to accumulation of type I procollagen due to a delayed secretion [3]. However, further studies are needed to find which mechanisms are involved in the nearly full lack of *FKBP10* transcripts and why the leaky expression of this gene is not sufficient to reduce the clinical presentations. Moreover, the functional analysis of *FKBP10* gene in the mutant cells and its effect on gene expression of type I collagen should be elucidated.

In conclusion, in current study a novel splice-acceptor mutation in *FKBP10* gene was identified which segregated with the disease phenotype (an autosomal recessive form of OI). The mutation is a private mutation without previous reports or documents in the checked variation databases such as ExAC, GenomAD, Kaviar, GME, ESP, and 1000Genomes. In addition, the effects of this mutation on exclusion of exon 8 was confirmed using Sanger sequencing, RT-PCR and Real-Time PCR on RNA samples derived from skin biopsy of the proband and carrier members. This study can prove the strong link between *FKBP10* gene and collagen fiber arrangement in bone tissue.

## Additional file

Additional file 1: Material and Methods, two supplementary tables, Description of data: Detail description of Fibroblast culture, Isolation of PBMCs and Quantitative RT-PCR. **Table S1.** List of all genes involved in Osteogenesis imperfecta. **Table S2.** Bioinformatics analysis statistics. (DOCX 25 kb)

## Abbreviations

MAF: Minor allele frequency
NGS: Next generation sequencing
NMD: Nonsense-mediated decay mechanisms
NVD: Normal vaginal delivery
OI: Osteogenesis imperfecta
RT-PCR: Reverse transcriptase polymerase chain reaction
VUS: Variation of unknown significance
WES: Whole exome sequencing
